# ERRATUM: GH modulates hepatic epidermal growth factor signaling in the mouse

**DOI:** 10.1530/JOE-09-0372e

**Published:** 2024-06-24

**Authors:** Lorena González, Ma Eugenia Díaz, Johanna G Miquet, Ana I Sotelo, Diego Fernández, Fernando P Dominici, Andrzej Bartke, Daniel Turyn

**Affiliations:** 1 Departamento de Química Biológica, Facultad de Farmacia y Bioquímica, Instituto de Química y Fisicoquímica Biológicas (UBA-CONICET), Universidad de Buenos Aires, Buenos Aires, Argentina; 2 Cátedra de Bioquímica Humana, Facultad de Medicina (UBA), Buenos Aires, Argentina; 3 Geriatrics Research, Departments of Internal Medicine and Physiology, School of Medicine, Southern Illinois University, Springfield, Illinois, USA

The authors and journal apologise for an error in the above paper, which appeared in volume 204, part 3, pages 299–309. The error relates to Fig. 6 given on page 306, in which the STAT5 Western-blotting image included in panel C does not correspond to the experiment shown. The corrected Fig. 6 is given in full below.

**Figure 6:**
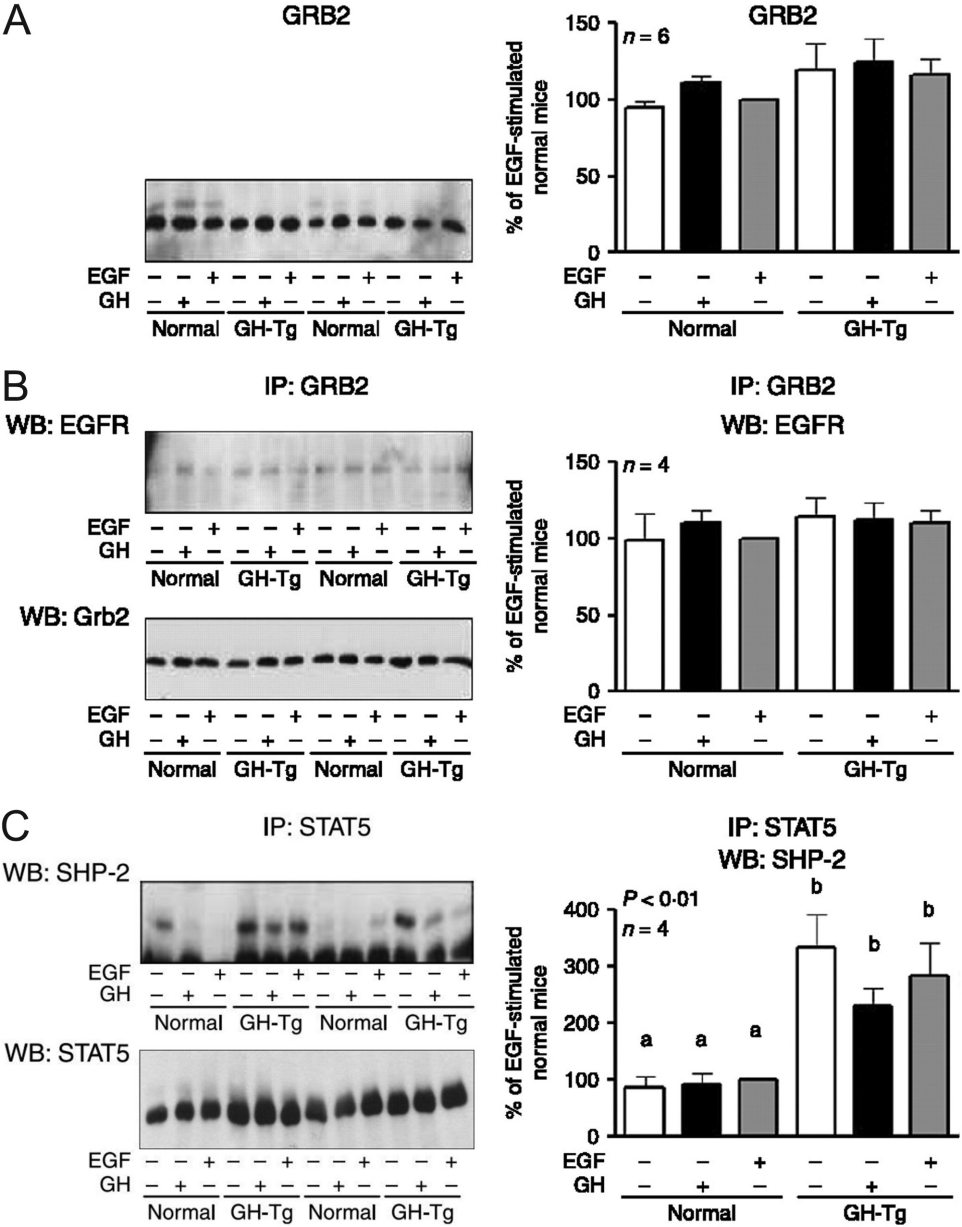
GRB2 content, GRB2/EGFR co-immunoprecipitation, and STAT5/SHP-2 co-immunoprecipitation in normal and GH-overexpressing transgenic mice. Normal and PEPCK-bGH transgenic mice (GH-Tg) were injected i.p. with saline, GH (2.5  mg/kg BW), or EGF (2  mg/kg BW), killed after 7.5 or 10  min, respectively, and the livers were removed. Equal amounts of solubilized liver protein were separated by SDS-PAGE and subjected to immunoblot analysis with anti-Grb2 (A) or were immunoprecipitated with anti-Grb2 or anti-STAT5 antibodies, separated by SDS-PAGE and subjected to immunoblot analysis with anti-EGFR and anti-Grb2 (B) or anti-SHP2 and anti-STAT5 (C) respectively. Representative results of immunoblots are shown. The amount of immunoprecipitated protein was determined by immunoblotting with the corresponding antibody. Quantification was performed by scanning densitometry and expressed as percent of values measured for EGF-stimulated normal mice. Data are the mean ± s.e.m. of the indicated number of subsets (*n*) of different individuals. Different letters denote significant difference at *P* < 0.05. Representative results from two samples per experimental condition are shown.

